# Tualang Honey Attenuates Noise Stress-Induced Memory Deficits in Aged Rats

**DOI:** 10.1155/2016/1549158

**Published:** 2016-03-28

**Authors:** Khairunnuur Fairuz Azman, Rahimah Zakaria, Che Badariah Abdul Aziz, Zahiruddin Othman

**Affiliations:** ^1^Department of Physiology, School of Medical Sciences, Universiti Sains Malaysia, Kubang Kerian, 16150 Kota Bharu, Kelantan, Malaysia; ^2^Department of Psychiatry, School of Medical Sciences, Universiti Sains Malaysia, Kubang Kerian, 16150 Kota Bharu, Kelantan, Malaysia

## Abstract

Ageing and stress exposure may lead to memory impairment while oxidative stress is thought to be one of the underlying mechanisms involved. This study aimed to investigate the potential protective effects of Tualang honey supplementation on memory performance in aged rats exposed to noise stress. Tualang honey supplementation was given orally, 200 mg/kg body weight for 28 days. Rats in the stress group were subjected to loud noise, 100 dB(A), 4 hours daily for 14 days. All rats were subjected to novel object recognition test for evaluation of memory performance. It was observed that the rats subjected to noise stress exhibited significantly lower memory performance and higher oxidative stress as evident by elevated malondialdehyde and protein carbonyl levels and reduction of antioxidant enzymes activities compared to the nonstressed rats. Tualang honey supplementation was able to improve memory performance, decrease oxidative stress levels, increase brain-derived neurotrophic factor (BDNF) concentration, decrease acetylcholinesterase activity, and enhance neuronal proliferation in the medial prefrontal cortex (mPFC) and hippocampus. In conclusion, Tualang honey protects against memory decline due to stress exposure and/or ageing via enhancement of mPFC and hippocampal morphology possibly secondary to reduction in brain oxidative stress and/or upregulation of BDNF concentration and cholinergic system.

## 1. Introduction

It is well known that ageing leads to a progressive loss of cognitive function, especially in spatial and working memory [1]. There is abundant evidence that such age-associated alterations in cognition are related to decreased function of cholinergic neurons in the hippocampus and cortex [2–4]. Survival and functional maintenance of cholinergic neurons are dependent upon nerve growth factors (NGF) including brain-derived neurotrophic factor (BDNF) [5–7]. In regions such as the hippocampus and cerebral cortex, NGF and BDNF supplementation protects neurons against experimentally induced damage from procedures [8, 9]. Neurotrophin supplementation also appears to have functional consequences for learning and memory and effectively ameliorates memory deficits in aged rats [10–12].

Noise exposure exceeding 90 dB has been reported to be a source of stressor [13]. Working and reference memory error increased significantly following the noise stress exposure, 100 dB(A)/4 h per day for 30 days, when compared to control animals [14]. Acute and chronic exposure to noise can produce excessive free radicals which may attack protein, nucleic acids, and lipid membranes thereby disrupting normal cellular functions [15]. Exposure to long-term oxidative stress in brain tissue has been shown to cause reductions in learning [16, 17] and memory [18, 19]. Several studies have shown that noise induces hippocampal dependent memory deficits and volumetric reduction in all layers of hippocampal field [15–17, 20].

Malaysia Tualang honey is a wild pure multifloral honey produced by Asian rock bee species (*Apis dorsata*), which builds hives on the branches of Tualang tree (*Koompassia excelsa*) located mainly in the rainforest of northern Peninsular Malaysia. Honey contains significant antioxidant activities as well as choline and acetylcholine which are essential for brain function and as neurotransmitters [21–25]. Recent studies reported that honey is one of the natural preventive therapies of both cognitive decline and dementia, as it possesses antioxidant properties and it enhances the brain's cholinergic system [26]. Correspondingly, another study reported that consumptions of honey may improve spatial memory in middle aged rats compared to those fed sucrose or sugar-free diet [27]. In closely related studies, it was demonstrated that Tualang honey was able to improve memory performance in stressed ovariectomized rats [28] and postmenopausal women [29]. Therefore, it is hypothesized that Tualang honey could revert oxidative stress by removing the reactive oxygen species that were produced during the noise stress and thus prevent oxidative damage in memory-related brain areas particularly in aged rats.

## 2. Material and Methods

### 2.1. Experimental Animals

A total of 48 male Sprague-Dawley rats (16 months old, weighing 600–800 g) were purchased from Sterling Ascent Sdn Bhd (Malaysia). The rats were maintained in standard polypropylene cages (40 × 25 × 16 cm) under a reversed 12-hour light/dark cycle (lights off at 0800 h) at a consistent room temperature of 27 ± 1°C in the laboratory of Animal Research and Service Centre, Universiti Sains Malaysia. The rats received commercial rat chow food pellets (Gold Coin Ltd., Malaysia) and water* ad libitum*. Rats were allowed to acclimatize to the holding room for 24 h before the behavioural procedures. The procedures in this study were approved by Animal Ethics Committee of Universiti Sains Malaysia (USM/Animal Ethics Approval/2013(85)(444)), in accordance with the internationally accepted principles for laboratory animal use and care.

### 2.2. Honey Supplementation

The Tualang honey used was from a single batch honey supplied by Federal Agricultural Marketing Authorities (FAMA), Malaysia. The honey was filtered by FAMA to remove solid particles, concentrated in an oven at 40°C, and evaporated to achieve a water content of about 20%. It was then subjected to *γ* irradiation at 25 kGy at Steril Gamma (M) Sdn. Bhd. (Selangor, Malaysia) for sterilization and bottled 230 g per jar. The final concentration of the bottled Tualang honey was 1.3 g/mL. Tualang honey at 200 mg/kg body weight/day [28, 30] was administered via oral gavage 14 days prior to stress procedure and the treatments were continued throughout the 14 days of stress procedure. The Tualang honey was freshly dissolved in 1 mL of distilled water prior to administration. Control groups received an identical volume of distilled water as placebo for the same period of time.

### 2.3. Experimental Design

The animals were randomly assigned to the following groups (*n* = 12): (i) nonstressed with placebo, (ii) nonstressed with Tualang honey, (iii) stressed with placebo, and (iv) stressed with Tualang honey. All rats were subjected to novel object recognition test for cognitive performance evaluation and killed by decapitation upon completion of the test. Individual body weight was recorded weekly using electrical balance. Blood samples (10 mL) were collected immediately. All blood samples were left to clot for 2 h prior to centrifugation for 15 min at 4000 rpm. Approximately 3 mL of serum was collected and stored at −20°C until assay. The brain of each animal was quickly harvested and weighed. The right brain hemisphere was homogenated (10% w/v) in ice-cold 0.1 M phosphate-buffered saline at pH 7.4. The homogenate was then centrifuged at 10000 ×g for 10 minutes and kept at −80°C until being analyzed. The left brain hemisphere was immediately fixed in 10% formalin for histological study.

### 2.4. Noise Stress Exposure

The animals of the test groups ((iii) and (iv)) were exposed to white noise for 4 hours (0900–1300 h) daily for 14 days. Noise was recorded from the generator and amplified by speakers in a separate room. Speakers were located 30 cm above the cages. The noise level was set at 100 dB(A) and intensity was measured by a sound level meter CENTER 325 (range: 80–130 dB(A), accuracy: +1.5 dB(A), made in Taiwan). Sound levels were verified in the centre of the cage before each exposure and varied by less than 1 dB(A) in the space the cage occupied. The control groups ((i) and (ii)) were kept in the same room for the same period of time without switching on the noise.

### 2.5. Novel Object Recognition Test

The test was carried out in a separate room that was ventilated, soundproofed, and maintained at a constant temperature (27 ± 1°C). The animals were brought into the test room 1 hour before the test commenced to minimize the arousal caused by the transference. The test was performed during the active period of the animals (dark phase) between the hours of 0900–1400. All the animals were tested in a random order. The trained observer remained blind to the treatment group of the rats until scoring was completed.

The test employed was similar to that described elsewhere [31]. The test uses the natural preference for novel object displayed by rats and normally used to assess cognitive alterations associated with ageing, genetic manipulations, or drug treatments. The chamber was an open field apparatus (60 × 60 × 30 cm). Training sessions were conducted on two successive days during which they were allowed to explore the arena for 10 min each day. In the training session, two identical sample objects were placed in the field in a symmetrical position about 10 cm away from the wall. After the two successive training sessions, testing/retention sessions were conducted. The retentions sessions consisted of two sessions, that is, short-term memory and long-term memory in which the retention interval for short-term memory and long-term memory was 2 hours and 24 hours after the last training session, respectively. In the retention session, rats were placed back in the same field, wherein one of the familiar objects used in the training session was replaced by a novel object and the rats were allowed to explore for 5 minutes.

All objects consisted of plastic toys and had a height of about 5 cm. Objects presented similar textures, colours, and sizes but distinctive shapes. The location of objects was alternated with each new animal; it was approximately placed in 50% trials in the right side and 50% in the left side of the field. Between tests, the objects were cleaned with 10% ethanol solution to mask any olfactory cues. Exploration was defined as sniffing or touching the object with the nose. Sitting on the object was not considered as exploration.

Total exploration times of the familiar and novel objects were recorded and used to calculate a discrimination index [time spent with novel object − time spent with familiar object]/[total time exploring both objects]. The discrimination index can range from −1 to 1 wherein −1 indicates complete preference for the familiar object, 0 signifies no preference for either object, and 1 indicates complete preference for the novel object. Increased preference to novel object was interpreted as successful memory retention for the familiar object. An absence of any difference in the exploration of the two objects was interpreted as memory deficit.

### 2.6. Serum Corticosterone and Adrenocorticotropic Hormone (ACTH) Levels

Serum levels of corticosterone and ACTH were measured by enzyme-linked immunosorbent assay (ELISA) kits using polyclonal antibody specific for corticosterone (LDN Labor Diagnostika Nord GmbH & Co. KG, Nordhorn, Germany) and monoclonal antibody specific for ACTH (Cloud-Clone Corp., Houston, USA).

### 2.7. Brain Oxidative Stress Markers

The evaluation of oxidative stress in the brain homogenates was performed by measuring the levels of plasma malondialdehyde (MDA) and protein carbonyl (PCO). The concentration of MDA was analyzed using commercially available kits from Northwest Life Sciences Specialties, Washington, USA, whereas the level of PCO was determined by commercially available kits from Cayman Chemical, Michigan, USA.

### 2.8. Brain Antioxidant Enzymes Activities

The activities of superoxide dismutase (SOD), glutathione peroxidase (GPx), and glutathione reductase (GR) in the brain homogenates were measured using commercially available kits from Northwest Life Sciences Specialties, Washington, USA. Commercially available kits from Bioassay Systems, California, USA, and Oxford Biomedical Research, Michigan, USA, were used to determine the activities of catalase (CAT) and the total antioxidant capacity, respectively.

### 2.9. Acetylcholinesterase (AChE) Activity Measurement

Commercially available assay kit from Bioassay Systems, California, USA, was used. The assay is based on an improved Ellman method, in which thiocholine produced by the action of AChE forms a yellow colour with 5,5′-dithiobis(2-nitrobenzoic acid). The intensity of the product colour, measured at 412 nm, is proportionate to the enzyme activity in the sample.

### 2.10. Brain-Derived Neurotrophic Factor (BDNF) Concentration Measurement

BDNF concentration was measured by ELISA kit from Boster Biological Technology Co., California, USA. A monoclonal antibody from mouse specific for BDNF and the assay Avidin-Biotin-Peroxidase complex was used to bind the detection antibody. HRP substrate 3,3′,5,5′-tetramethylbenzidine (TMB) was used to visualize HRP enzymatic reaction which produces a blue colour product that changed into yellow after adding acidic stop solution. The density of yellow colour, measured at 450 nm, is proportionate to the BDNF concentration in the sample.

### 2.11. Protein Concentration

Following homogenization, an aliquot was removed from each brain sample to determine its protein concentration using commercially available kits from Bioassay Systems, California, USA. Briefly, protein concentration was quantified by comparing the colorimetric intensity of the reaction product from each sample with a series of protein standards. All antioxidant activities and oxidative stress markers levels were normalized to their total protein concentration in the sample in order to account for possible differences in protein concentrations between samples.

### 2.12. Histopathological Analysis

The left brain hemispheres were embedded in paraffin wax, cut into 5 *μ*m thick coronal sections using a rotary microtome, and mounted on slides, followed by Nissl staining, which was performed according to the standard procedure. The slides were observed under a light microscope and images were captured to visualize the arrangement of pyramidal neurons in the medial prefrontal cortex (mPFC) and each hippocampal region: CA1, CA2, CA3, and DG. The Nissl-positive cells were counted at different magnifications using High Definition Medical Image Analysis Program (analySIS docu 5.0, Münster, Germany). The mean of two fields was taken as the number of Nissl-positive cells for each section and the mean of four sections was taken as the number of Nissl-positive cells of each group. Cells which had a shrunken or unclear body with surrounding empty spaces were excluded.

### 2.13. Statistical Analysis

All analyses were performed using IBM SPSS statistics software (version 20.0). Statistical data are expressed as mean ± SEM, and a result was deemed to be statistically significant if *P* < 0.05. Two-way analyses of variance (ANOVA) were utilized to examine the main effects of stress (nonstressed versus stressed) and honey treatment (placebo versus honey) on the memory performance, antioxidant enzymes activities, oxidative stress markers, stress hormone levels, and the number of Nissl-positive cells in the mPFC and hippocampal regions. After confirming the normality of data and the homogeneity of variance of data, the significance of the differences between the means of the test and control studies was established by one-way analysis of variance (ANOVA) coupled with* post hoc* Tukey HSD test.

## 3. Results

### 3.1. Effects of Tualang Honey on Body Weight

**Figure 1:**
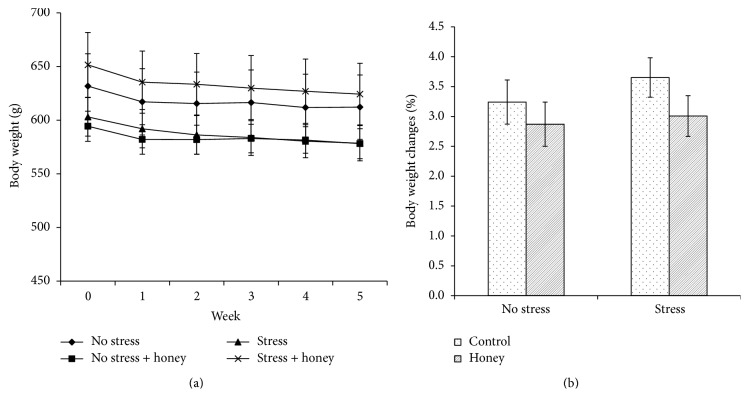
Effects of stress and honey treatment on (a) body weight and (b) percentage of body weight changes. The values are expressed as mean ± SEM.

Mean body weights of all groups over five weeks of experimental period were illustrated in Figure 1(a). Percentage of body weight changes was calculated as [(final body weight − initial body weight)/initial body weight] × 100%. There was no significant difference in the percentage of body weight changes between all groups (Figure 1(b)).

### 3.2. Effects of Tualang Honey on Serum Corticosterone and ACTH Levels

A significant effect of stress (*F*_1,22_ = 8.24, *P* < 0.01) was observed on the serum ACTH level whereas significant effects of honey treatment were observed on the serum corticosterone (*F*_1,20_ = 3.93, *P* < 0.05) and ACTH (*F*_1,22_ = 11.82, *P* < 0.01) levels (Table 1). There was no significant stress-treatment interaction (*F*_3,19_ = 2.01, *P* > 0.05) in corticosterone level while significant stress-treatment interaction (*F*_3,21_ = 6.92, *P* < 0.01) in ACTH level was observed. Stress exposure significantly (*P* < 0.05) increases ACTH level while honey treatment significantly (*P* < 0.05) decreases corticosterone and ACTH levels in the stressed rats.

### 3.3. Effects of Tualang Honey on Memory Performance

**Figure 2:**
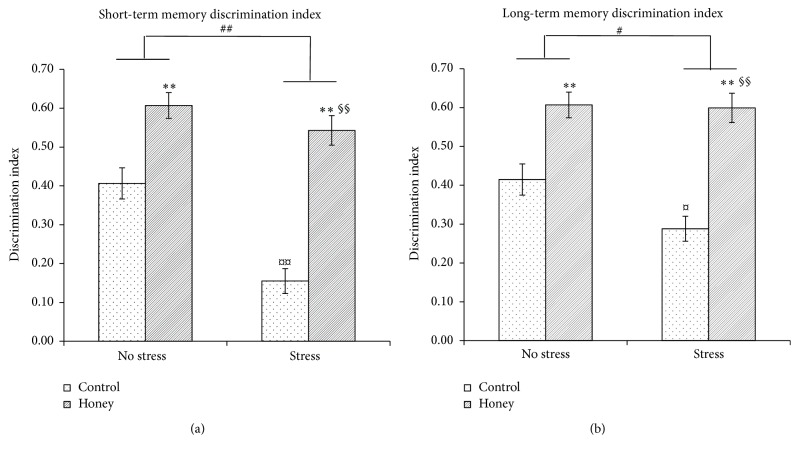
Effects of stress and honey treatment on mean discrimination index ratio of (a) short-term memory and (b) long-term memory. The values are expressed as mean ± SEM. Significant main effects of loud noise stress (^#^ *P* < 0.05; ^##^ *P* < 0.01). Significant main effects of honey treatment (^*∗*^ *P* < 0.05; ^*∗∗*^ *P* < 0.01). Significant difference between no stress and stress control (^¤^ *P* < 0.05; ^¤¤^ *P* < 0.01). Significant difference between stress control and stress treated with honey (^§^ *P* < 0.05; ^§§^ *P* < 0.01).

Two-way ANOVA revealed significant effects of stress on short-term memory (*F*_1,27_ = 16.06, *P* < 0.001) and long-term memory (*F*_1,28_ = 3.65, *P* < 0.05), indicating that stress was associated with memory deficit (Figures 2(a) and 2(b)). Interestingly, there were significant effects of honey treatment on short-term memory (*F*_1,27_ = 55.59, *P* < 0.001) and long-term memory (*F*_1,28_ = 47.29, *P* < 0.001). Stressed rats supplemented with honey showed significantly (*P* < 0.001) higher mean discrimination index in both short- and long-term memory compared to stressed control rats indicating better memory performance. There were significant stress-treatment interactions in short-term memory (*F*_3,26_ = 33.33, *P* < 0.001) and long-term memory (*F*_3,27_ = 19.40, *P* < 0.001).

### 3.4. Effects of Tualang Honey on Brain Oxidative Status

The effects of Tualang honey administration on brain tissue damage index were evaluated as the content of MDA and PCO and the antioxidative defence systems such as SOD, GPx, GR, CAT, and total antioxidant capacity (Table 2). Two-way ANOVA revealed significant effects of stress on the levels of MDA (*F*_1,27_ = 12.52, *P* < 0.01), PCO (*F*_1,13_ = 10.66, *P* < 0.01), SOD (*F*_1,34_ = 9.60, *P* < 0.01), CAT (*F*_1,34_ = 6.31, *P* < 0.05), and total antioxidant capacity (*F*_1,29_ = 8.03, *P* < 0.01). Stress exposure increases oxidative stress as evident by elevated levels of MDA and PCO, while the activity of antioxidant enzymes as well as the total antioxidant capacity was reduced. There was significant effect of honey treatment on the activity of SOD (*F*_1,34_ = 10.22, *P* < 0.01). There were significant stress-treatment interactions in the levels of MDA (*F*_3,26_ = 5.76, *P* < 0.01), PCO (*F*_3,12_ = 6.40, *P* < 0.01), SOD (*F*_3,37_ = 7.50, *P* < 0.01), and total antioxidant capacity (*F*_3,28_ = 3.23, *P* < 0.05). One-way ANOVA confirms that stressed rats treated with honey possessed significantly lower MDA (*P* < 0.05) and PCO (*P* < 0.05) levels and significantly higher (*P* < 0.001) SOD activity compared to stressed control rats.

### 3.5. Effects of Tualang Honey on Brain AChE Activity

**Figure 3:**
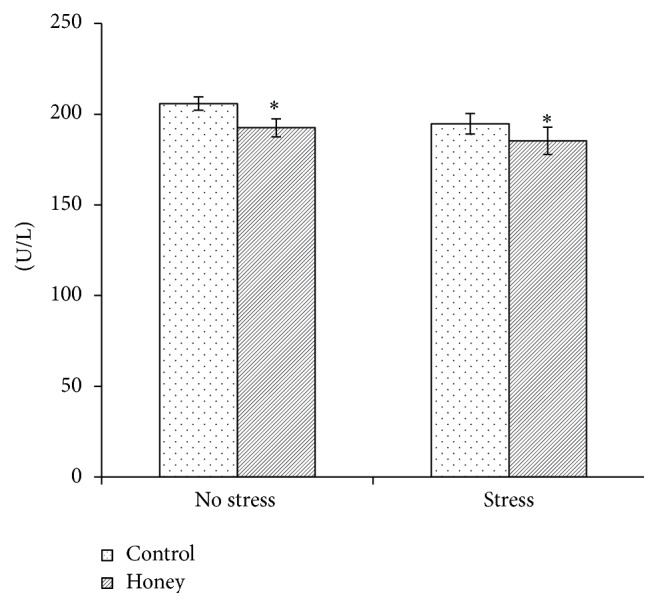
Effects of stress and honey treatment on acetylcholinesterase activity. The values are expressed as mean ± SEM. Significant main effects of honey treatment (^*∗*^ *P* < 0.05).

An inhibitory effect of honey treatment was observed on the AChE activity (*F*_1,20_ = 3.93, *P* < 0.05) (Figure 3). No significant effect of stress and stress-treatment interaction was observed.

### 3.6. Effects of Tualang Honey on BDNF Concentration

**Figure 4:**
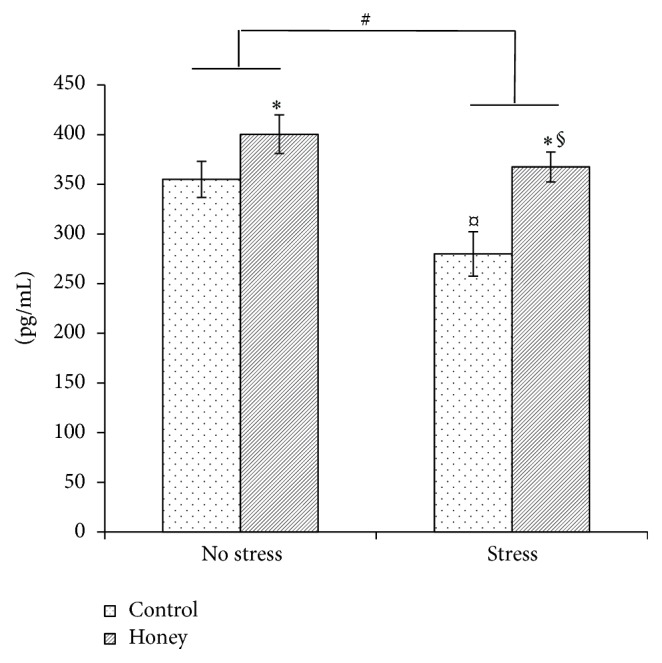
Effects of stress and honey treatment on BDNF concentration. The values are expressed as mean ± SEM. Significant main effects of loud noise stress (^#^ *P* < 0.05). Significant main effects of honey treatment (^*∗*^ *P* < 0.05). Significant difference between no stress and stress control (^¤^ *P* < 0.05). Significant difference between stress control and stress treated with honey (^§^ *P* < 0.05).

A significant effect of stress (*F*_1,22_ = 8.24, *P* < 0.01) and honey treatment (*F*_1,22_ = 11.82, *P* < 0.01) was observed on the BDNF concentration (Figure 4). Significant interaction between stress and treatment (*F*_3,21_ = 6.92, *P* < 0.01) was evident. Stress exposure significantly (*P* < 0.05) decreases BDNF concentration whereas honey treatment significantly (*P* < 0.05) increases BDNF concentration in the stressed rats.

### 3.7. Effects of Tualang Honey on the Number of Nissl-Positive Cells in mPFC and Hippocampal Regions

Two-way ANOVA revealed significant effect of stress on the number of Nissl-positive cells in CA2 hippocampal region (*F*_1,9_ = 20.14, *P* < 0.01). Significant effects of honey treatment were observed on the number of Nissl-positive cells in mPFC (*F*_1,9_ = 22.94, *P* < 0.01), CA2 (*F*_1,9_ = 29.08, *P* < 0.001), CA3 (*F*_1,9_ = 6.72, *P* < 0.05), and DG hippocampal regions (*F*_1,9_ = 6.93, *P* < 0.05) (Table 3). There were significant stress-honey interactions on the number of Nissl-positive cells in mPFC (*F*_3,8_ = 7.32, *P* < 0.05) and CA2 hippocampal region (*F*_3,8_ = 21.69, *P* < 0.001). One-way ANOVA shows that stressed control rats possessed significantly (*P* < 0.05) lower number of Nissl-positive cells in the CA2 hippocampal region than the nonstressed control. Stressed rats treated with Tualang honey have a significantly (*P* < 0.05) higher number of Nissl-positive cells in the mPFC and CA2 hippocampal region compared to stressed control rats.

### 3.8. Effects of Tualang Honey on Arrangement of Nissl-Positive Cells in mPFC and Hippocampal Regions

**Figure 5:**
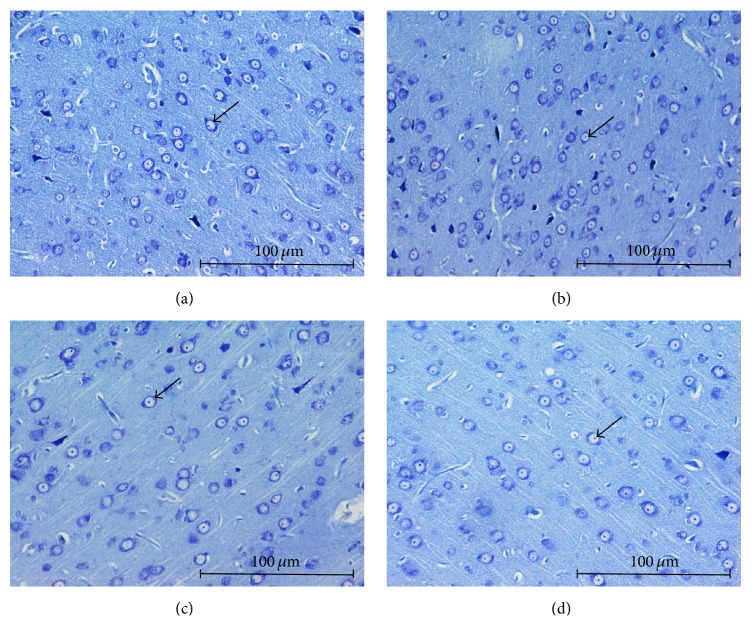
Arrangement of mPFC pyramidal neurons among groups; (a) no stress, (b) no stress + honey, (c) stress, and (d) stress + honey. The arrows indicate the cells of interest (Nissl staining ×200, scale bar: 100 *μ*m).

**Figure 6:**
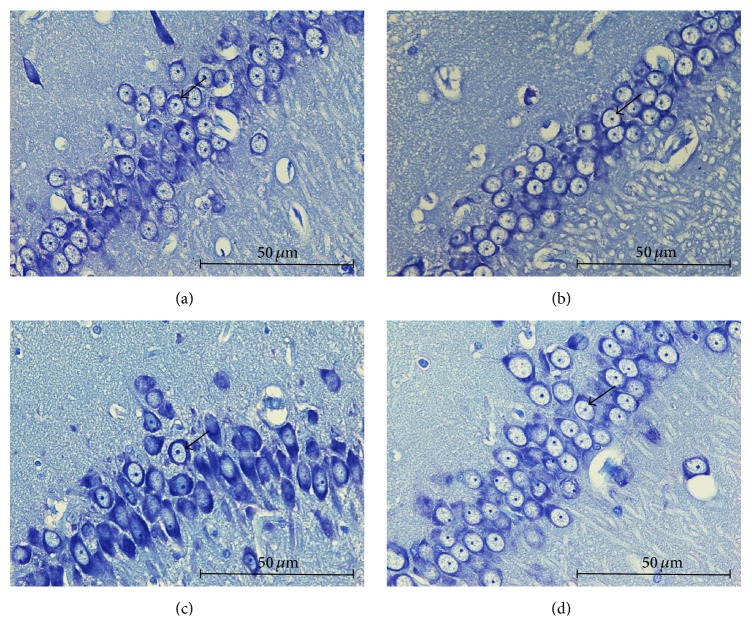
Arrangement of hippocampal CA2 pyramidal neurons among groups; (a) no stress, (b) no stress + honey, (c) stress, and (d) stress + honey. The arrows indicate the cells of interest (Nissl staining ×400, scale bar: 50 *μ*m).

Stressed control group exhibited moderately shrunken neuronal cell bodies with cytoplasmic vacuolation (Figures 5(c) and 6(c)). The arrangement of pyramidal neurons was sparse and the Nissl substance was decreasing or dissolving. In contrast, stressed group treated with honey exhibited abundant pyramidal neurons, the architecture of these neurons was preserved, and Nissl substances in the cytoplasm were clearly visible (Figures 5(d) and 6(d)). Arrangement of pyramidal neurons of the stress group treated with honey appeared quite similar to the nonstressed group.

## 4. Discussion

In the present study, no significant differences in the change of body weight between groups were observed suggesting that stress and honey treatment did not cause substantial effect on the body weight. In addition, we demonstrated that aged male rats exposed to noise stress exhibited higher corticosterone and ACTH levels than the nonstressed rats. Accumulating evidence suggests that the hypothalamic-pituitary-adrenal (HPA) axis is altered by ageing [32, 33]. The pituitary, hypothalamus, and hippocampus express glucocorticoid receptors and may all be putative sites for glucocorticoid-mediated negative feedback on the pituitary-adrenal axis [34]. Various studies demonstrated that aged rats exhibit a loss of glucocorticoid receptors in the hippocampus and thus are impaired in their ability to terminate a stress response [35–37], resulting in accumulation of stress hormones following stress exposure.

Loss of glucocorticoid receptors in the hippocampus would obviate glucocorticoid-mediated negative feedback inhibition of corticotropin-releasing factor (CRF) expression and secretion, resulting in increased HPA activity, a further downregulation of glucocorticoid receptors, and, potentially, a loss of hippocampal neurons [36]. Correspondingly, we demonstrated that the stressed rats possessed lower number of pyramidal neurons in the hippocampus as well as in the mPFC compared to the nonstressed rats. Similarly, Manikandan et al. [14] reported significant decreases in the dendritic count in the CA1 and CA3 regions of rat hippocampus after noise stress exposure, 100 dB(A)/4 h per day for 30 days, while Sharifabad and Sabahi [20] reported that chronic noise exposure, 40 dB(A)/1 h per day for 90 days, reduces the volume of CA3 and DG hippocampal subregions.

Along with that, the stressed rats demonstrated significantly lower BDNF concentration than the nonstressed rats. Previous studies reported that stress exposure may cause reduction of BDNF expression in the hippocampus and paraventricular nucleus (PVN) [38–41]. The reduction of hippocampal BDNF expression induced by stress may also contribute to the hippocampal neuronal loss [42] as observed in this study. The reduced BDNF concentration and neuronal loss in the mPFC and hippocampus may have manifested their effects as memory impairment. In the present study, we demonstrated that aged male rats exposed to noise stress were impaired on both short- and long-term memory as assessed in the novel object recognition test. It is widely accepted that noise exposure is a stressful environmental stimulus and has been previously shown to impair cognition such as the acquisition of memory, consolidation, and recall [43]. The observed adverse effects of noise exposure on recognition memory are in agreement with previous studies [14, 44].

Interestingly, our data showed that supplementation of Tualang honey was able to improve both short- and long-term memory in the stressed rats. The histopathological results revealed an increase in the number of neuronal cells in mPFC, CA2, CA3, and DG hippocampal regions in the groups treated with Tualang honey, which could be interpreted as tissue preservation and maintenance of the ability to retain information. Earlier studies by our research team revealed that Tualang honey supplementation was able to improve memory performance in young rats exposed to noise stress [45] as well as in ovariectomized rats exposed to social instability stress [28]. It is suggested that Tualang honey, a phytoestrogen, mediated its neuroprotective effects through its antioxidant properties and via upregulation of BDNF expression [46] and augmentation of choline acetyltransferase and acetylcholinesterase activities in specific brain areas [47]. Honey also modulates ACTH and corticosterone levels following noise stress either by suppressing HPA mobilization in response to stress or by facilitating elevated plasma corticosterone and ACTH levels back to baseline following the termination of the stress. Future studies should investigate these possible antistress properties of honey and also measure glucocorticoid receptors following honey supplementation.

In conjunction with that, we demonstrated that the aged stressed rats treated with Tualang honey exhibited significantly higher BDNF concentration than the untreated stressed rats. Numerous reports have also demonstrated that NGF administration improved learning and memory impairments in aged rats, coupled with a partial reversal of cholinergic neuron atrophy [10, 12]. Flavonoids have been demonstrated to enhance glutamate signaling, which leads to continuous activation of cell survival signaling pathways, such as PKC and PI3K in the brain cells, triggers gene and protein expression, which most likely is CREB-dependent, and then causes a more stable long-term potential [48–50]. Since BNDF is regulated by CREB, it is believed that the flavonoids contents in the honey may have mimicked the above mechanism thus exhibiting its neuroprotective effect.

Our finding on AChE activity following noise stress was in contrast with earlier report by Manikandan et al. [14] which showed significant increase in AChE activity in animals exposed to noise stress. This discrepancy might be contributed to the difference in age of rats used. However, our result revealed that Tualang honey supplementation was able to decrease AChE activity in aged rats with or without noise stress exposure. Decrease in AChE activity might lead to an increase in acetylcholine in the synaptic cleft and a consequent increase in the cholinergic activity, which in turn leads to memory improvement. Numerous other studies have reported the AChE inhibitory effects of plant extracts, for instance,* Morinda citrifolia *L. (Noni fruit) [51],* Rosmarinus officinalis *L. (Rosemary leaf) [52],* Astragali Radix, *and* Salviae Miltiorrhizae Radix *[53], where all of those extracts exhibited memory enhancing abilities. Thus, it is suggested that the memory enhancing ability of Tualang honey is partly due to the inhibition of brain AChE activity.

Studies have shown that noise exposure causes an increase in reactive oxygen species (ROS), resulting in oxidative stress. The negative effects of noise on cell structure and function could be, at least in part, mediated by this increase in ROS [54]. For example, in an investigation of the effects of moderate-intensity white noise exposure on learning and memory of mice, the results showed evidence of oxidative damage in the auditory cortex and hippocampus [55]. In yet another study, Manikandan et al. [56] found increased MDA levels in different areas of the brain after 30 days of 100 dB white noise exposure. Our results are in accordance with these findings where exposure to noise was shown to induce oxidative stress as evident by elevated levels of MDA and PCO in the brain of the stressed rats. However, Manikandan et al. [14, 56] reported contradictory results especially on the SOD level which could possibly be due to better oxidative stress compensation in adults rats compared to aged rats.

In order to protect cells from oxidative damage, aerobic metabolism generally depends on a stringent control of ROS by antioxidants. Several antioxidant enzymes within the framework of the antioxidant defence system in our body are SOD, GPx, GR, and CAT. In our study, it was observed that the levels of SOD, CAT, and total antioxidant capacity of the stressed rats were significantly lower than the nonstressed rats. The results we obtained are similar to those of other studies [57, 58]. The decrease in antioxidant enzymes activities might be explained by the consumption of antioxidant enzymes developed by the increase in MDA and PCO preceded by stress exposure.

Phytochemical screening of honey reveals that it contains flavonoids, phenolic acids, ascorbic acid, *α*-tocopherol, and carotenoids [24, 59, 60]. It is widely known that these compounds possessed antioxidant activities. It was previously reported that the total phenolic content of Tualang honey was 251.7 ± 7.9 mg gallic acid/kg honey and total antioxidant activity was 322.1 ± 9.7 (*μ*M Fe(II)) whereas the antiradical activity was 41.30 ± 0.78 (% inhibition) [61]. Correspondingly, in the current study, it was observed that Tualang honey significantly improved brain oxidative status as shown by elevated antioxidant enzymes activities and reduced oxidative markers. Other studies have also demonstrated that honey is able to increase antioxidant enzymes activities and ameliorate oxidative stress in plasma and other tissues such as kidney and pancreas [62–66]. Thus, it is assumed that the improvement of memory performance in the stressed rats by Tualang honey is also partly due to its antioxidant capacity which is attributed to the aforementioned antioxidant compounds. This is in keeping with studies which have demonstrated that dietary antioxidants improved cognitive performance in clinical studies [67, 68] as well as in animals [69–71].

## 5. Conclusion

In conclusion, the memory enhancing effects of Tualang honey could act via enhancement of cholinergic system and mPFC and hippocampal morphology possibly secondary to reduction in brain oxidative stress and/or upregulation of BDNF concentration. The presence of several bioactive compounds in honey, particularly flavonoids and phenolic acids, might be responsible for these effects. Further studies have to be conducted to evaluate honey biological activity as well as its toxicity in order to support its potential use as an alternative therapy to protect against memory deterioration due to stress exposure and/or ageing in both males and females.

## Figures and Tables

**Table 1:** Effects of stress and honey treatment on serum corticosterone and adrenocorticotropic hormone levels.

	No stress	No stress + honey	Stress	Stress + honey
Corticosterone level (ng/mL)	68.75 ± 2.88	65.68 ± 2.35^*∗*^	73.82 ± 2.31	65.90 ± 1.84^*∗*§^
Adrenocorticotropic hormone level (pg/mL)	95.90 ± 4.10	82.43 ± 4.35^*∗*^	113.78 ± 4.72^#¤^	91.75 ± 3.40^*∗*§^

The values are expressed as mean ± SEM. Significant main effects of loud noise stress (^#^ *P* < 0.05). Significant main effects of honey treatment (^*∗*^ *P* < 0.05). Significant difference between no stress and stress control (^¤^ *P* < 0.05). Significant difference between stress control and stress treated with honey (^§^ *P* < 0.05).

**Table 2:** Effects of stress and honey treatment on brain oxidative status.

	No stress	No stress + honey	Stress	Stress + honey
MDA (*μ*M/mg protein)	7.03 ± 0.44	7.31 ± 0.32	8.76 ± 0.27^#¤^	7.88 ± 0.20^§^
PCO (nmol/mg protein)	2.37 ± 0.40	3.15 ± 0.66	5.17 ± 0.36^#¤^	3.73 ± 0.22^§^
SOD activity (U/mg protein)	9.68 ± 1.87	12.39 ± 1.12^*∗*^	4.83 ± 0.73^#¤^	9.87 ± 0.68^*∗*§§^
GPx activity (mU/mg protein)	52.71 ± 8.74	52.38 ± 9.53	42.91 ± 7.15	43.84 ± 9.17
GR activity (mU/mg protein)	80.93 ± 12.29	75.82 ± 5.55	57.61 ± 6.75	72.77 ± 6.63
CAT activity (mU/mg protein)	4660.80 ± 156.59	4748.84 ± 246.92	4240.81 ± 226.86^#^	4149.37 ± 116.38
Total antioxidant capacity (*μ*M Trolox equivalents/mg protein)	0.05 ± 0.00	0.05 ± 0.00	0.04 ± 0.00^#^	0.04 ± 0.00

The values are expressed as mean ± SEM. Significant main effects of loud noise stress (^#^ *P* < 0.05). Significant main effects of honey treatment (^*∗*^ *P* < 0.05). Significant difference between no stress and stress control (^¤^ *P* < 0.05). Significant difference between stress control and stress treated with honey (^§^ *P* < 0.05; ^§§^ *P* < 0.01).

**Table 3:** Effects of stress and honey treatment on the number of Nissl-positive cells in mPFC and hippocampal regions.

	No stress	No stress + honey	Stress	Stress + honey
mPFC/0.04 mm^2^	14.43 ± 0.75	20.07 ± 1.88^*∗*^	14.13 ± 0.76	18.63 ± 0.45^*∗*§^
CA1 region/0.01 mm^2^	24.44 ± 1.06	31.11 ± 4.78	27.22 ± 1.13	27.78 ± 2.13
CA2 region/0.01 mm^2^	23.56 ± 2.47	33.89 ± 4.64^*∗∗*^	19.33 ± 2.60^#¤^	24.89 ± 4.13^*∗∗*§^
CA3 region/0.01 mm^2^	10.22 ± 1.28	17.22 ± 1.64^*∗*^	14.56 ± 0.80	15.56 ± 1.44^*∗*^
DG region/0.01 mm^2^	34.56 ± 3.80	38.22 ± 3.61^*∗*^	33.22 ± 2.15	39.11 ± 2.21^*∗*^

The values are expressed as mean ± SEM. Significant main effects of loud noise stress (^#^ *P* < 0.05). Significant main effects of honey treatment (^*∗*^ *P* < 0.05; ^*∗∗*^ *P* < 0.01). Significant difference between no stress and stress control (^¤^ *P* < 0.05). Significant difference between stress control and stress treated with honey (^§^ *P* < 0.05).
